# Psychometric Properties of an Instrument for Assessing University Administrators’ Knowledge on Gender-Based Violence

**DOI:** 10.1590/0034-7167-2022-0770

**Published:** 2023-12-04

**Authors:** Elisabeth Meloni Vieira, Maria Paula Panúncio-Pinto, Deíse Camargo Maito, Maria Carmen Martinez

**Affiliations:** I Universidade de São Paulo. São Paulo, São Paulo, Brazil; II Universidade de São Paulo. Ribeirão Preto, São Paulo, Brazil; III Universidade do Estado de Minas Gerais. Ituiutaba, Minas Gerais, Brazil; IV WAF Informática & Saúde. São Paulo, São Paulo, Brazil

**Keywords:** Validation Study, Factor Analysis, Statistical, Psychometrics, Gender-Based Violence, Workplace Violence, Estudio de Validación, Análisis Factorial, Psicometría, Violencia de Género, Violencia Laboral, Estudo de validação, Análise Fatorial, Psicometria, Violência Baseada em Gênero, Violência no Ambiente de Trabalho

## Abstract

**Objective:**

To evaluate the factorial structure of the instrument measuring university administrators’ knowledge of gender-based violence.

**Methods:**

This cross-sectional methodological study was conducted from August to November 2020 with 101 university administrators. Data on demographic and functional characteristics were collected, and the “QUEST VBG UNIV” instrument was applied. Descriptive analysis was performed, the structure of the questionnaire was assessed using exploratory factor analysis (EFA), and the stability of the factors was verified through ORION and FDI tests.

**Results:**

Of the original 38 items across the 4 sections of the questionnaire, 19 were retained within 2 factors, with appropriate factor loadings. Factor 1 had an explained variance of 15.69%, and Factor 2 had an explained variance of 9.10%. The reliability was deemed satisfactory (ORION > 0.900, FDI > 0.900).

**Conclusions:**

The questionnaire presented a valid and reliable factorial structure for measuring knowledge about gender-based violence, thereby representing a suitable option for situational assessments in universities.

## RESUMO

**Objetivo:**

Avaliar a estrutura fatorial do instrumento de medição do conhecimento de gestores sobre violência de gênero na universidade.

**Métodos:**

Estudo transversal de caráter metodológico, realizado no período de agosto a novembro de 2020 com 101 gestores universitários. Foram coletados dados sobre características demográficas e funcionais, e o instrumento “QUEST VBG UNIV” foi aplicado. Realizou-se análise descritiva, avaliação da estrutura do questionário usando análise fatorial exploratória (AFE), e verificação da estabilidade dos fatores pelos testes ORION e FDI.

**Resultados:**

Dos 38 itens originais das 4 seções do questionário, 19 foram retidos em 2 fatores, com cargas fatoriais adequadas. O Fator 1 teve variância explicada de 15,69%, e o Fator 2 de 9,10%. A confiabilidade foi considerada satisfatória (ORION > 0,900, FDI > 0,900).

**Conclusões:**

O questionário apresentou estrutura fatorial válida e confiável para mensurar o conhecimento sobre violência de gênero, representando uma opção adequada para avaliações situacionais em universidades.

## RESUMEN

**Objetivo:**

Evaluar la estructura factorial del instrumento que mide el conocimiento de los administradores universitarios sobre la violencia de género.

**Métodos:**

Estudio metodológico transversal se llevó a cabo de agosto a noviembre de 2020 con 101 administradores universitarios. Se recogieron datos sobre características demográficas y funcionales, y se aplicó el instrumento “QUEST VBG UNIV”. Se realizó un análisis descriptivo, se evaluó la estructura del cuestionario utilizando análisis factorial exploratorio (AFE), y se verificó la estabilidad de los factores a través de las pruebas ORION y FDI.

**Resultados:**

De los 38 ítems originales en las 4 secciones del cuestionario, se retuvieron 19 en 2 factores, con cargas factoriales adecuadas. El Factor 1 tuvo una varianza explicada del 15.69%, y el Factor 2 una varianza explicada del 9.10%. Se consideró que la fiabilidad era satisfactoria (ORION > 0.900, FDI > 0.900).

**Conclusiones:**

El cuestionario es una herramienta válida y confiable para medir el conocimiento sobre la violencia de género en las universidades.

## INTRODUCTION

Violence against women, or Gender-Based Violence (GBV), is an escalating public health issue worldwide and must be recognized as a socio-historical phenomenon, complex in its multicausality and its multiple consequences. This type of violence is present in the university context, making it crucial to measure the understanding of Gender-Based Violence within the university, especially from the perspective of administrators, who are responsible for university policies.

The WHO recognizes violence against women as a global health problem of epidemic proportions^(1)^. GBV refers to situations that affect women because they are women, and its various manifestations range from subtle forms, which may be overlooked as violence in daily relations (such as sexist jokes and minor disqualifications based on gender stereotypes), to terribly concrete forms, such as sexual violence and femicide^(2)^.

Situations of discrimination and gender-based violence are also present in the academic university context, affecting the personal and professional development of female students, employees, and teachers, as various studies point out^(3-6)^. In the Brazilian university context, work on the topic of GBV in academia has been produced^(7-9)^, notably after the repercussion of the report from the Parliamentary Inquiry Commission of the Legislative Assembly of the State of São Paulo, Brazil^(10)^, which investigated violence within the context of the Universities of São Paulo, calling them to confront these forms of violence in the university everyday life^(11-12)^.

Much has been discussed, since the last three decades of the twentieth century, about the challenge that higher education institutions must face: to train not only technically competent professionals but also individuals with humanism, ethics, and political commitment in building a better society that welcomes diversity and promotes the rights of all and equity^(13)^. Thus, the university is conceived as a space for the encounter of various forms of knowledge, where solutions to social issues are sought^(14)^. In this context, it’s important to recognize that the university, as a social institution, reflects the way society functions and, as a subset of it, is affected by the dominant ideology. Gender-based violence is structural and is universally expressed, produced, and reproduced, regardless of socioeconomic, educational, and cultural conditions^(15)^. Therefore, various types of violence against women present in society in general are reproduced in the context of higher education, from subtle daily discriminations to more concrete forms of violence, such as sexual violence.

At the University in focus, where all stages of this study were carried out, 2/3 of the teaching staff are men, and hirings over the past decades perpetuate this pattern, which is reflected in its scientific output. Subjected to a gender bias, it remains a predominantly male institution, as the highest-ranking and most powerful positions are dominated by men, who, in turn, have a higher H index in publications, increasing their chances of advancement in the academic career^(16)^.

In 2019, the “Interactions” survey^(17)^, with the participation of 13,377 undergraduate and post graduate students, showed that, although the majority of students consider the university a less discriminatory environment than society in general, 26% rated the institution as very sexist; 26% as very racist; 11% as very LGBTphobic, and 56% as very elitist. In this study, violence based on gender, race, and social class was also evidenced. An example is the moral violence suffered by women and men, from social markers of differences: of the black homo or bisexual women who participated in the survey, 52% suffered moral violence, that is, more than triple the percentage of heterosexual white men who suffered the same type of violence (17%). This is an example of how violence is interconnected with social markers, as per the concept of intersectionality^(18)^.

At the University in question, policies were established to confront GBV after the mobilization of student and teacher collectives^(19)^. In 2016, a partnership between the university and UN Women was created^(20)^. This context of mobilization also resulted in the creation of special commissions to deal with violence, such as CAV-Women^(21)^.

In this scenario, with the institutional commitment to confront all forms of discrimination and GBV, the study that originated the questionnaire, whose process of evaluating measurement properties is reported here, was developed.

## OBJECTIVE

To evaluate the factorial structure of an instrument that measures university administrators’ knowledge of gender-based violence.

## METHODS

### Ethical Aspects

The study adhered to both national^(22)^ and international ethical guidelines and was approved by the Research Ethics Committee *of Hospital das Clínicas da Faculdade de Medicina de Ribeirão* Preto/SP. The committee’s opinion is attached to this submission.

Free and Informed Consent was obtained from all study participants online, via the completion of an online form held by the researchers.

### Study Design, Period, and Location

This cross-sectional methodological study, guided by the STROBE tool, was carried out at São Paulo University (USP) in the municipality of Ribeirão Preto from August to November 2020.

### Population and Sample

The study population comprised 259 university administrators on a campus of the subject university who occupied 283 positions, given that it’s possible for one person to hold more than one position. Inclusion criteria included being involved in the implementation of university policy due to participation in collegiate bodies or administrative positions and having held disciplinary power in teaching units over the past four years. Exclusion criteria included inability to make contact (7 people were in this condition) and being in close proximity to the research team (4 people were in this condition).

The sample was non-probabilistic, using convenience sampling. A total of 248 staff members-both teaching (directors, chairpersons of statutory commissions, course coordinators, department heads and their deputies) and non-teaching (academic, administrative, financial technical assistants, and representatives from their respective categories in congregations and technical-administrative councils of the units)-were identified and invited to participate. The 101 administrators who responded to the emails and completed the instruments during the specified data collection period were included in the study.

### Study Protocol

The instrument used was named “Questionnaire for Assessing Institutional Agents’ Knowledge about Gender-Based Violence at the University”, or in its abbreviated form, “QUEST VBG UNIV”. The questionnaire was part of a larger study conducted to broaden the understanding of how the University in question handles gender-based violence (GBV), aiming to identify the University’s institutional agents’ knowledge of gender-based violence on the said campus (23). The research was developed in two stages. The first consisted of 17 qualitative interviews with key informants (students, teachers, and employees) conducted with a semi-structured script. The analysis of these interviews served as the basis for the construction of the questionnaire, which was applied in the subsequent quantitative stage.

The questionnaire was specifically constructed for this study, as scientific literature provides similar instruments for investigating student perceptions of violence, but not university administrators’^(24-25)^. Other studies investigated intimate partner violence among university students, which was also not the focus of our study^(26-27)^.

The questionnaire contained the following groups of variables:

1. “Professional characterization,” with six questions about the current position, career position, and previous positions. These questions are objective and can be modified according to the characteristics of the location and population of each new application. They were not included in the factorial analysis.
2. “Knowledge about violence in the university environment,” with 10 questions referencing categories identified in the qualitative stage and in the Interactions research^(17)^. The responses to each question in this section were tallied in a score, where each correct answer corresponds to one point, and incorrect answers and “don’t know” options were not scored. Part “a” of question 10 is only descriptive and does not score, being excluded from the factorial analysis.
3. “Opinion on violence at the university,” with 6 statements based on qualitative interviews and classified according to a Likert scale. To obtain the participants’ opinion, a figure was displayed with the options: “strongly agree,” “agree,” “neither agree nor disagree,” “disagree,” and “strongly disagree.” The “strongly agree” option counts as 1 point, “agree” as 2 points, “neither agree nor disagree” as zero, “disagree” as 3, and “strongly disagree” as 4 points.
4. “Experience with violence and discrimination at the university,” a section composed of 11 questions, covering the experience in the current management position, as well as the experience before it (witnessed or received reports of different types of violence). Question 17, on a Likert scale, counts 4 points for the “frequently” option, 3 for “sometimes”, 2 for “rarely”, and 1 point for “never”. Question 21, on a Likert scale, counts 1 point for the “frequently” option, 2 points for “sometimes”, and 3 points for both “rarely” and “never”. For the remaining questions, each affirmative response receives 1 point, and the sum of the categories defines a score: 0 to 6 points for questions 18, 19, 20, 22, 23, and 25; 0 to 7 points for questions 24 and 26; 0 to 5 points for question 27. Parts “a” and “b” of question 23, parts “a” and “b” of question 26, and question 27 are only descriptive, do not score, and are excluded from the factorial analysis.
5. “Knowledge about procedures related to gender-based violence and ways to confront it,” a section composed of 12 questions with true or false, yes or no, and multiple-choice questions. Correct answers are based on current Brazilian legislation^(28)^, literature^(18,29-30)^, and administrative norms applicable to USP^(21,31-33)^. Each correct answer scored a positive point; errors and “don’t know” options were not scored.
6. “Sociodemographic information of participants,” with 13 questions such as age, sex, sexual orientation, religion, marital status, number, and sex of children. These questions are objective and can be modified according to the characteristics of the location and population of each new application. They were not included in the factorial analysis.

### Study Protocol Modification and Analysis

The questionnaire underwent modifications inspired by a literature review of Rowling’s work ^(34)^ and a pre-test with five university professors, two of whom are specialists in the field of violence. The results from the first version of this questionnaire’s application have been published^(35)^, and the complete original questionnaire can be found in Supplemental Material 1. In addition, questions about demographic characteristics were incorporated, including sex, sexual orientation, age range, race/color, religion, marital status, and the presence of children. Data collection was conducted face-to-face on the Google Meets platform by two trained interviewers. Data collection was supervised on a weekly basis, facilitating critique of the questionnaire and control of the field.

### Data Analysis and Statistics

Initially, a descriptive analysis was conducted using absolute and relative values to characterize the sample. Based on the literature standards of the American Educational Research Association / American Psychological Association / National Council on Measurement in Education (2014)^(35)^, the COSMIN Consensus - COnsensus-based Standards for the selection of health Measurement INstruments^(36)^, and other authors proposals^(37)^, the measurement properties were evaluated through construct validity, investigating the questionnaire’s internal structure, and through the instrument’s reliability, probing its internal consistency and stability.

The evaluation of the internal structure verifies the degree to which the items and components of an instrument reflect the dimensionality of the construct to be measured, based on the proposed score interpretations^(35-37)^. To assess the factorial structure of the QUEST VBG UNIV, Exploratory Factor Analysis (EFA) was carried out, based on the procedures proposed by Lorenzo-Seva and Ferrando^(38)^ and Damásio^(39)^. Of the 58 original questions, 38 were included in the analysis, 6 were excluded because they are pertinent to the professional characterization, 13 to sociodemographic characterization, and one due to its purely descriptive nature. A Pearson correlation matrix was used, along with a robust factorial analysis technique, and a robust diagonally weighted least squares extraction method (RDWLS). Parallel Analysis with a random permutation of the observed data was implemented for the determination of the number of factors to retain, and the rotation used was Robust Promin.

The suitability of applying EFA to the dataset was evaluated by Bartlett’s test of sphericity and the Kaiser-Meyer-Olkin (KMO) measure, while the model’s adequacy was assessed via the Root Mean Square Error of Approximation (RMSEA), Comparative Fit Index (CFI), Tucker-Lewis Index (TLI), and Minimum Fit Function Chi Square (χ2/gl) indices.

The questionnaire’s unidimensionality was appraised through the unidimensional congruence (UniCo), explained common variance (ECV), and mean of item residual absolute loadings (MIREAL) indicators. Reliability pertains to a test’s capability to consistently reproduce results over time, across space, or during test procedure replications^(37-38)^. The internal consistency and stability of the achieved factors were evaluated via the composite reliability index - FC, the Overall Reliability of fully-Informative prior Oblique N-EAP scores (ORION), the Factor Determinacy Index (FDI), and the H-Latent and H-Observed (generalized h index - H Index) estimates^(38)^. Additionally, an assessment of the questionnaire items’ internal consistency was conducted using McDonald’s omega coefficient.

### Software Utilized

Data collection and storage were conducted through the REDCap application. The data were then exported to Excel and subsequently to STATA version 14 for consistency verification, recoding, and descriptive data analysis. Factor Program version 11.05.01 was utilized for conducting the EFA, and the Composite Reliability Calculator from The Statistical Mind website was used for calculating the composite reliability index. For the calculation of the omega coefficient, JASP software, version 0.16.4, was used.

## RESULTS

In regards to the characteristics of the managers who made up the study population (as shown in the table in Appendix 2), we observed that women constituted 50.5% of the participants. Additionally, 63.4% were 50 years of age or older, 81.2% self-identified as white, 52.5% identified as Christian (either Catholic or Evangelical), 83.2% were married or in a stable relationship, and 77.2% had children. With respect to their professional history, 50.5% had been employed at the university for up to 18 years, 45.5% held management positions (as directors, vice-directors, or chairs of statutory committees), 37.6% had been in a management position for more than two years, 86.1% had prior management experience, and 84.2% were part of the teaching staff (as associate professors, full professors, or endowed chairs).

The EFA was initially conducted with the four factors anticipated in the questionnaire structure (knowledge about violence and VBG, experience with violence and VBG, opinion about violence and VBG, and knowledge about procedures) and their respective 38 questions. However, the parallel analysis did not confirm this projected structure, indicating that the instrument consists solely of two factors: knowledge and experience.

**Figure 1:**
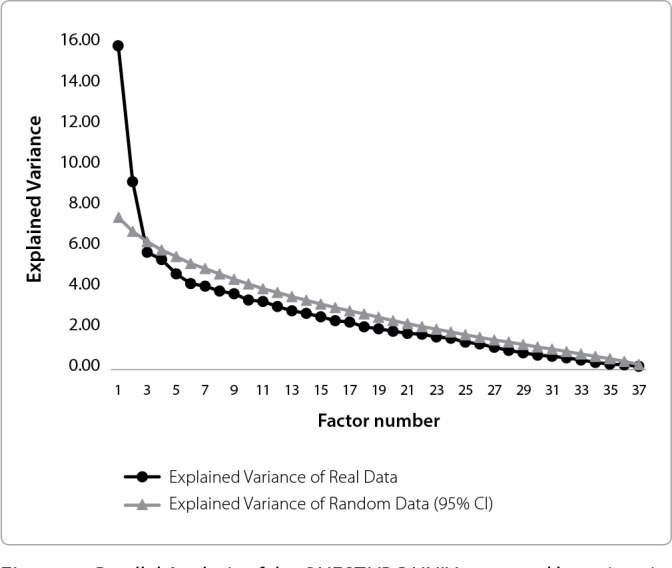
Parallel Analysis of the QUEST VBG UNIV answered by university managers, *Universidade de São Paulo*, from September to November 2020, Ribeirão Preto, São Paulo, Brazil

We subsequently executed another EFA model with the two identified factors. Bartlett’s test of sphericity (995.9, df=703, p<0.001) demonstrated that the correlation matrix was favorable, and the Kaiser-Meyer-Olkin (KMO - 0.622) index suggested a mediocre interpretability of the item correlation matrix. The parallel analysis ratified the two factors as the most representative for the data, which were renamed Current Knowledge/Experience (Factor 1) and Previous Knowledge/Experience (Factor 2) in relation to violence at the university. Factor 1 accounted for 15.69% of the variance, while Factor 2 accounted for 9.10% of the variance. The data are illustrated in Figure 1.

The factor loadings of the items on the two factors and the stability indices are presented in Table 1. Of the original 38 items from the 4 sections of the questionnaire, 19 were retained in the 2 obtained factors, with 11 items in Factor 1 and 8 items in Factor 2. The retained items demonstrated adequate factor loadings. No pattern of cross-loadings was found, with factor loadings above 0.300 or below -0.300 in more than one factor.

**Table 1:** Factor structure of the University Violence Questionnaire answered by university managers, *Universidade de São Paulo*, from September to November 2020, Ribeirão Preto, São Paulo, Brazil

Question	Abbreviated Content^*^	Factor 1^**^	Factor 2^**^
1	The university environment is the space defined within the campuses.	-0.016	0.059
2	The university is a place where gender-based violence occurs.	-0.085	-0.245
3	Universities demand specific policies to address violence.	-0.030	-0.226
4	What is the frequency of violence in the university environment?	-0.410	-0.129
5	What is the frequency of sexism and sexual discrimination in the university environment?	-0.436	-0.132
6	Manifestation of violence in the university context.	0.219	0.188
7	Sexual assault on a vulnerable person.	-0.121	-0.121
8	Frequency of parties as scenarios for sexual assaults on vulnerable individuals.	-0.295	-0.147
9	Frequency of spontaneous reports of experiencing violence.	-0.057	-0.265
10	Groups of people most likely to experience violence.	-0.244	0.020
11	Cases of violence outside the campus should not be considered as university violence.	0.362	0.097
12	Body painting, toll collection, haircutting, and the use of props should not be considered hazing.	-0.223	0.333
13	Hazing is prohibited and no longer occurs.	0.197	0.144
14	Certain jokes between seniors and freshmen should not be considered hazing.	-0.122	0.316
15	Men and women are treated equally by everyone.	0.399	0.243
16	The university is prepared to combat discrimination.	0.362	0.124
17	In the position you hold, how frequently are you informed about a violence situation?	-0.001	-0.883
18	In the position you hold, how many groups of people report violence?	0.051	0.881
19	In the position you hold, how many types of violence reports exist?	-0.020	0.913
20	In the position you hold, how many types of discrimination reports exist?	0.056	0.625
21	How frequently do you consult the General Regulations?	0.232	-0.167
22	Before the position you held, how many groups of people reported violence?	0.909	-0.007
23	Before the position you held, how many types of violence reports existed?	0.803	0.106
24	Before the position you held, how many types of discrimination did you experience?	0.489	-0.062
25	Before the position you held, how many groups of discriminated people did you witness?	0.848	-0.220
26	Before the position you held, how many types of discrimination did you witness?	0.832	-0.084
27	When you were a student, how many instances of hazing violence were there?	0.299	-0.001
28	Gender-based violence refers exclusively to women.	-0.078	-0.110
29	Percentage of women in Brazil who have experienced violence.	-0.096	0.087
30	Daily human rights violations in the academic community are not the responsibility of the university and its managers.	0.038	-0.108
31	Mechanisms provided in the Regulations do not guarantee protection and support for victims of violence.	-0.067	-0.263
32	Violence against a woman at a fraternity party extends beyond the university environment.	0.217	-0.155
33	Disciplinary power in case of a complaint of gender-based violence at University X.	-0.118	0.299
34	The appropriate conduct of a manager in cases of gender-based violence.	-0.035	0.324
35	An investigative commission to examine a situation of violence against women must maintain a gender perspective.	0.035	0.028
36	Institutional responsibility in addressing situations of gender-based violence according to legislation.	-0.120	-0.026
37	The conduct of members of an investigative commission.	0.307	-0.262
38	Women in situations of violence have the right to comprehensive care, and it is the institution's duty to provide it.	0.054	-0.308
Composite Reliability		0.844	0.814
Orion		0.929	0.936
FDI		0.964	0.967

* *Content abbreviated due to space. The complete content of the questions is presented in Appendices 1 and 3.*

* ^*^ Factor 1 = Current Knowledge/Experience Factor 2 = Previous Knowledge/Experience

*Note: ORION = Overall Reliability of fully-Informative prior Oblique N-EAP scores, FDI = Factor Determinacy Index*

The evaluation of the model’s adequacy demonstrated that the factorial structure exhibited suitable adjustment indices or ones very close to the ideal: RMSEA=0.051, CFI=0.947, TLI=0.941, and χ^2^=668.94 with df=628 and p=0.125. The unidimensionality of the scale was refuted by the UniCo (0.713), ECV (0.634), and MIREAL (0.232) indicator values, while the evaluation of the instrument’s reliability indicated that, for both factors, the composite fidelity indices were favorable (>0.800), as were the ORION (>0.900), FDI (>0.900), and the latent and observed H index estimates (>0.900).

Additionally, the instrument’s internal consistency was evaluated using McDonald’s omega coefficient. In Table 2, it can be observed that when all 38 items of the instrument are considered, the result of the coefficient is unsatisfactory (ω=0.580). When the evaluation is repeated, considering the items retained in each factor in the EFA, it can be seen that the results are more favorable, with ω=0.770 in factor 1 and ω=0.666 in factor 2. However, the analysis revealed that some items are negatively correlated with the scale, suggesting that they should be reversed. These items are the same ones that exhibited a negative factorial load in the EFA: in factor 1, these are items “4. Frequency of violence in the university environment” and “5. Frequency of sexism and sexual discrimination in the university environment.” When the scoring of these items is reversed, the results become more favorable, with ω=0.858 in factor 1 and ω=0.805 in factor 2.

**Table 2:** McDonald’s Omega values per question of the University Violence Questionnaire answered by university managers, *Universidade de São Paulo*, from September to November 2020, Ribeirão Preto, São Paulo, Brazil

Items	Abbreviated Content^*^	All items	Factor 1	Factor 1 with inverted scoring^**^	Factor 2^**^	Factor 2 with inverted scoring^**^
1	The university environment is the space defined within the campuses.	0.578	..	..	..	..
2	The university is a place where gender-based violence occurs.	0.596	..	..	..	..
3	Universities demand specific policies to address violence.	0.586	..	..	..	..
4	What is the frequency of violence in the university environment?	0.639	0.825	0.856	..	..
5	What is the frequency of sexism and sexual discrimination in the university environment?	0.631	0.810	0.854	..	..
6	Manifestation of violence in the university context.	0.559	..	..	..	..
7	Sexual assault on a vulnerable person.	0.600	..	..	..	..
8	Frequency of parties as scenarios for sexual assaults on vulnerable individuals.	0.633	..	..	..	..
9	Frequency of spontaneous reports of experiencing violence.	0.598	..	..	..	..
10	Groups of people most likely to experience violence.	0.586	..	..	..	..
11	Cases of violence outside the campus should not be considered as university violence.	0.559	0.767	0.858	..	..
12	Body painting, toll collection, haircutting, and the use of props should not be considered hazing.	0.585	..	..	0.678	0.816
13	Hazing is prohibited and no longer occurs.	0.567	..	..	..	..
14	Certain jokes between seniors and freshmen should not be considered hazing.	0.579	..	..	0.678	0.817
15	Men and women are treated equally by everyone.	0.562	0.758	0.851	..	..
16	The university is prepared to combat discrimination.	0.571	0.762	0.854	..	..
17	In the position you hold, how frequently are you informed about a violence situation?	0.597	..	..	0.738	0.767
18	In the position you hold, how many groups of people report violence?	0.565	..	..	0.460	0.716
19	In the position you hold, how many types of violence reports exist?	0.573	..	..	0.466	0.710
20	In the position you hold, how many types of discrimination reports exist?	0.560	..	..	0.589	0.776
21	How frequently do you consult the General Regulations?	0.581	..	..	..	..
22	Before the position you held, how many groups of people reported violence?	0.421	0.654	0.816	..	..
23	Before the position you held, how many types of violence reports existed?	0.386	0.710	0.834	..	..
24	Before the position you held, how many types of discrimination did you experience?	0.552	0.759	0.856	..	..
25	Before the position you held, how many groups of discriminated people did you witness?	0.489	0.701	0.834	..	..
26	Before the position you held, how many types of discrimination did you witness?	0.453	0.694	0.826	..	..
27	When you were a student, how many instances of hazing violence were there?	0.560	..	..	..	..
28	Gender-based violence refers exclusively to women.	0.587	..	..	..	..
29	Percentage of women in Brazil who have experienced violence.	0.589	..	..	..	..
30	Daily human rights violations in the academic community are not the responsibility of the university and its managers.	0.582	..	..	..	..
31	Mechanisms provided in the Regulations do not guarantee protection and support for victims of violence.	0.597	..	..	..	..
32	Violence against a woman at a fraternity party extends beyond the university environment.	0.575	..	..	..	..
33	Disciplinary power in case of a complaint of gender-based violence at University X.	0.585	..	..	..	..
34	The appropriate conduct of a manager in cases of gender-based violence.	0.572	..	..	0.672	0.819
35	An investigative commission to examine a situation of violence against women must maintain a gender perspective.	0.583	..	..	..	..
36	Institutional responsibility in addressing situations of gender-based violence according to legislation.	0.594	..	..	..	..
37	The conduct of members of an investigative commission.	0.575	0.768	0.859	..	..
38	Women in situations of violence have the right to comprehensive care, and it is the institution's duty to provide it.	0.598	..	..	0.701	0.810
		0.580	0.770	0.858	0.666	0.805

* *Content abbreviated due to space. The full content of the questions is presented in Appendices 1 and 2.*

* ^*^ The following items (highlighted in bold) are negatively correlated with the scale and have had their scores inverted: factor 1 - items 4 and 5; factor 2 - items 17 and 38.

## DISCUSSION

This study aimed to conduct a psychometric validation of the ‘Questionnaire for the Evaluation of Institutional Agents’ Knowledge about Violence in the University’ (QUEST VBG UNIV). This instrument, constructed based on a literature review and a qualitative phase of the research, originally had a structure comprising four dimensions. However, these were not confirmed by the Exploratory Factor Analysis (EFA), with parallel analysis indicating two factors. Despite the ambiguous results regarding the adequacy of EFA for the sample data (with favorable results in Bartlett’s test and mediocre outcomes in Kaiser-Meyer-Olkin (KMO) measure), all other evaluations demonstrated a high degree of stability and reproducibility of the two factors obtained. These assessments included tests of composite reliability, ORION, Factorial Discriminant Index (FDI), and Latent and Observed H, internal consistency (using the omega coefficient), and a satisfactory or near-ideal model fit (as shown by RMSEA, Comparative Fit Index (CFI), Tucker-Lewis Index (TLI), and chi-square tests). Furthermore, the tests confirmed the non-unidimensional character of the questionnaire (UniCo, ECV, and MIREAL tests).

From the 38 items in the original composition of QUEST VBG UNIV, 19 were retained in the final questionnaire’s two factors (see Supplementary Material 2). Several potential elements may have led to the non-retention of items. One aspect is the similarity in the wording of some questions, although this was mitigated by the interview format of the questionnaire application, facilitated with interviewer guidance.

Another aspect is the structuring of the questions, some of which had ordinal responses while others were qualitative in nature. This could have interfered with normality and variances among the items, as qualitative variables did not necessarily follow an ordinal configuration. To minimize this, we used a robust factorial analysis and the Robust Diagonally Weighted Least Squares (RDWLS) extraction method, which considers the ordinal nature of the variables and adjusts the data to a normal distribution^(40-41)^.

Sample size is another consideration. There is no consensus on the ideal sample size for conducting factor analysis, with guidelines ranging from 100 to 250 subjects and/or a minimum of 5 to 20 observations per variable analyzed^(39,42)^. In this investigation, we performed 101 evaluations for 38 questions, resulting in a low ratio that may introduce some degree of imprecision into the results, a limitation of our study. Establishing an appropriate sample size is complex, as it depends on the number of measured variables, the strength of relationships between variables and factors, factor determination, and the quality of the instrument. However, it is generally agreed that factor analysis requires large samples because correlation coefficients fluctuate more in smaller samples, compromising the reliability of the factor analysis^(38,41-42)^. Caution should be used when incorporating samples from different populations, as the specific factors of one population may be obscured when grouped^(43)^. In this research, we limited the sample to a specific university campus, reducing this risk.

When considering the parameters from the literature^(39,43-44)^, the KMO test showed a mediocre shared variance among the variables. However, Bartlett’s test confirmed that the correlation matrix was not random. Therefore, the two factors composed of the 19 items obtained in this study could be considered plausible within a knowledge structure about GBV, which may depend on current and past experiences.

Another aspect that supports the appropriateness of the factorial structure obtained pertains to the stability tests. Composite reliability is a robust indicator used to determine item consistency, checking if all items consistently measure the same construct, based on factorial loads and measurement error variances^(45)^. The two factors obtained for QUEST VBG UNIV showed favorable results for internal consistency and absence of measurement error, supporting the association between knowledge and experience of GBV.

Furthermore, university managers do not associate GBV present in the university context with the recommendations outlined in the Maria da Penha Law^(23)^. With literature parameters^(46-47)^, internal consistency was also attested by the ORION and FDI indices, which showed favorable results in the precision of measuring factorial scores and the representation of the latent trait. The evaluation of internal consistency through McDonald’s omega coefficient yielded satisfactory results, particularly when the scores of items 4, 5, 17, and 38 were reversed due to their negative correlation with the scale. These items were also those with a negative factorial load in the EFA. Additionally, the bidimensional structure of the model showed a satisfactory fit for the studied sample, evidenced by the RMSEA, CFI, TLI, and chi-square indicators^(48-49)^, with unidimensionality refuted by the UniCo, ECV, and MIREAL indicators^(49)^.

### Study Limitations

The preliminary results of this study show evidence of appropriate measurement properties, supporting its use. However, the results also pointed to weaknesses in the instrument, especially concerning the response scoring scales. Furthermore, it is necessary to take into account that the sample was restricted to a single university campus, which prevents the establishment of the factor structure’s maintenance in other populations.

Given the scarcity of tools for measuring violence in the university environment, as well as the limitations of the instrument and the study design, it is suggested that new studies be conducted with other population groups and an expanded sample. It is also proposed that an evaluation be carried out with a committee of experts for the improvement of the instrument’s content, considering the theoretical framework used, the results obtained, and the characteristics of the instrument, especially in relation to item scoring.

### Contributions to the Field of Nursing and Public Health

The validation of this instrument can contribute to the diagnosis of reality regarding the operators of university policies: the confrontation of GBV in the university is intrinsically linked to the efficacy of university policies to combat violence, and the definition and execution of these policies are subordinate to the knowledge and experience of managers.

## CONCLUSIONS

The sum of the evaluations indicates that the QUEST VBG UNIV, in the configuration of the two retained factors, can be considered an adequate and reliable instrument to measure latent traits of knowledge about gender-based violence in the university. This instrument has applicability both in academic research on the subject and as a tool for situational diagnosis in university violence management programs.

## Supplementary Material

0034-7167-reben-76-06-e20220770-suppl01

0034-7167-reben-76-06-e20220770-suppl02

0034-7167-reben-76-06-e20220770-suppl03

## Data Availability

https://doi.org/10.48331/scielodata.ZIDWTD
