# Supplementary material for: Psychometric Properties of an Instrument for Assessing University Administrators’ Knowledge on Gender-Based Violence
Source: Rev Bras Enferm. 2023 Dec 4;76(6):e20220770. doi: 10.1590/0034-7167-2022-0770 (PMC10695057; doi:10.1590/0034-7167-2022-0770)
Supplement: 0034-7167-reben-76-06-e20220770-suppl01 [file 0034-7167-reben-76-06-e20220770-suppl01.pdf]

MATERIAL SUPLEMENTAR 1 – QUESTIONÁRIO ORIGINAL COMPLETO

Questionário para Avaliação do Conhecimento dos Gestores Universitários sobre Violência na Universidade

Parte 1. CARACTERIZAÇÃO PROFISSIONAL

QUESTÃO	CATEGORIAS DE RESPOSTAS	CÓDIGOS	PONTOS	OBSERVAÇÃO
A. Há quanto tempo você é docente/funcionário da universidade?	Até 18 anos	--	--	Não pontua no escore
	Mais de 18 anos	--	--	
B. Você ocupa algum cargo de gestão atualmente? Qual é?	Diretores, vice-diretores e presidentes de comissões estatutárias	--	--	Não pontua no escore
	Presidentes de CoCs, chefes e vice-chefes de departamento	--	--	
	Assistência técnica ou representante de funcionários na congregação/CTA	--	--	
C. Há quantos meses você está neste cargo?	Abaixo de um ano	--	--	Não pontua no escore
	De um ano até dois anos	--	--	
	Acima de dois anos	--	--	
D. Você já ocupou outros cargos de gestão antes deste	Frequentemente	--	--	Não pontua no escore
	Não sabe	--	--	
E. Qual foi o último cargo ocupado antes deste?	Chefe de departamento ou presidente de comissão	--	--	Não pontua no escore
	Membro de colegiado ou coordenação de curso	--	--	
	Outros cargos - funcionários	--	--	
	Outras comissões ou coordenação de serviços universitários	--	--	
F. Atualmente, qual posição você ocupa na carreira?	Professor Doutor 1 ou 2	--	--	Não pontua no escore
	Professor Associado 1, 2 ou 3	--	--	
	Professor titular	--	--	
	Servidor de qualquer nível/posição	--	--	

Parte 2. SEÇÃO CONHECIMENTO SOBRE VIOLÊNCIA NO AMBIENTE UNIVERSITÁRIO

QUESTÃO	CATEGORIAS DE RESPOSTAS	CÓDIGOS	PONTOS	OBSERVAÇÃO
1. O ambiente universitário é definido como o espaço delimitado apenas entre os muros dos campi	Verdadeiro	1	0	
	Falso	2	1	Categoria 2 (falso) é a resposta correta
	Não sabe	3	0	
2. Não existe evidência científica de que a universidade seja espaço onde ocorra violência de gênero atualmente	Verdadeiro	1	0	
	Falso	2	1	Categoria 2 (falso) é a resposta correta
	Não sabe	3	0	

3. A violência nas universidades é um fenômeno global que demanda políticas específicas	Verdadeiro<br>Falso<br>Não sabe	1<br>2<br>3	1<br>0<br>0	Categoria 1 (verdadeiro) é a resposta correta
4. Qual é a frequência da violência no ambiente universitário?	Frequentemente<br>Às vezes<br>Raramente<br>Nunca<br>Não sabe	1<br>2<br>3<br>4<br>5	0<br>1<br>0<br>0<br>0	Categoria 2 (às vezes) é a resposta correta
5. Qual é a frequência do machismo e da discriminação sexual no ambiente universitário?	Frequentemente<br>Às vezes<br>Raramente<br>Nunca<br>Não sabe	1<br>2<br>3<br>4<br>5	0<br>1<br>0<br>0<br>0	Categoria 2 (às vezes) é a resposta correta
6. Na sua opinião, a violência no âmbito universitário se manifesta entre quais pessoas?<br>Entre professores e estudantes<br>Entre estudantes e estudantes<br>Entre funcionários e funcionários<br>Entre professores e professores<br>Entre funcionários e estudantes<br>Entre funcionários e professores	Mesmas opções para todas as manifestações<br>Sim<br>Não<br>Não sabe	 1<br>2<br>3	 1<br>0	Categoria 1 (sim) é a resposta esperada para cada manifestação. A somatória dos pontos das categorias define um escore de 0 a 6 pontos
7. O estupro de vulnerável é a relação sexual com: (escolha somente uma das alternativas)	Pessoa menor de 14 anos	1	0	Categoria 4 (todas as alternativas) é a resposta correta
	Pessoa de qualquer idade que não tem condição de dar seu consentimento	2	0	
	Pessoa com deficiência mental	3	0	
	Todas as alternativas	4	1	
	Nenhuma das alternativas	5	0	
	Não sabe	6	0	
8. Qual a frequência de relatos de festas universitárias como cenários de estupros de vulneráveis?	Frequentemente<br>Às vezes<br>Raramente<br>Nunca<br>Não sabe	1<br>2<br>3<br>4<br>5	0<br>0<br>1<br>0<br>0	Categoria 3 (raramente) é a resposta correta
9. Na USP o relato espontâneo de ter sofrido qualquer tipo de violência atinge cerca de:	70% dos estudantes<br>40% dos estudantes<br>10% dos estudantes<br>Não sabe	1<br>2<br>3<br>4	0<br>1<br>0<br>0	Categoria 2 (40% dos estudantes) é a resposta correta
10. Você acha que alguns grupos de pessoas têm mais chances de sofrer violência na universidade?	Sim<br>Não<br>Não sabe	1<br>2<br>3	1<br>0<br>0	Categoria 1 (sim) é a resposta correta
10.a.Quais grupos<br>Mulheres	Mesmas opções para todos os grupos<br>Sim	 1		Essa questão não pontua, porque é

Pessoas não brancas	Não	0	vinculada à resposta afirmativa da questão anterior. É somente descritiva
Pessoas transexuais ou não binárias	Não sabe	2	
Pessoas homossexuais ou bissexuais			
Pessoas pobres			
Pessoas com deficiência			
Outro grupo. Qual?	Campo texto		

Parte 3. SEÇÃO OPINIÃO SOBRE VIOLÊNCIA NO AMBIENTE UNIVERSITÁRIO

QUESTÃO	CATEGORIAS DE RESPOSTAS	CÓDIGOS	PONTOS	OBSERVAÇÃO
Nas próximas seis perguntas agora eu vou ler algumas afirmações e você vai me dizer se você concorda plenamente, concorda, não concorda nem discorda, discorda e discorda plenamente. Vou mostrar as opções para você na tela.				
11. Casos de violência que possam acontecer em festas, repúblicas e torneios fora do campus, entre universitários, não devem ser considerados no âmbito da violência universitária.	Concordo plenamente	1	1	Quanto menor a pontuação, maior a tolerância à violência
	Concordo	2	2	
	Não concordo e nem discordo	3	0	
	Discordo	4	3	
	Discordo totalmente	5	4	
12. Pintar o corpo, fazer pedágio, cortar o cabelo e usar adereços não deve ser considerado trote pois não envolve violência entre os estudantes.	Concordo plenamente	1	1	Quanto menor a pontuação, maior a tolerância à violência
	Concordo	2	2	
	Não concordo e nem discordo	3	0	
	Discordo	4	3	
	Discordo totalmente	5	4	
13. O trote está proibido na universidade e, portanto, não ocorre mais.	Concordo plenamente	1	1	Quanto menor a pontuação, maior a tolerância à violência
	Concordo	2	2	
	Não concordo e nem discordo	3	0	
	Discordo	4	3	
	Discordo totalmente	5	4	
14. Brincadeiras entre veteranos e calouros, que incluem apelidos engraçados, pagar micos, fazer pedágios, etc., fazem parte da cultura universitária e não devem ser consideradas trotes.	Concordo plenamente	1	1	Quanto menor a pontuação, maior a tolerância à violência
	Concordo	2	2	
	Não concordo e nem discordo	3	0	
	Discordo	4	3	
	Discordo totalmente	5	4	
15. Homens e mulheres são tratados igualmente por todos no ambiente universitário.	Concordo plenamente	1	1	Quanto menor a pontuação, maior a tolerância à violência
	Concordo	2	2	
	Não concordo e nem discordo	3	0	
	Discordo	4	3	
	Discordo totalmente	5	4	
16. A universidade está preparada para enfrentar a discriminação de classe, sexo, gênero e raça entre seus membros.	Concordo plenamente	1	1	Quanto menor a pontuação, maior a tolerância à violência
	Concordo	2	2	
	Não concordo e nem discordo	3	0	
	Discordo	4	3	
	Discordo totalmente	5	4	

Parte 4. SEÇÃO EXPERIÊNCIA COM VIOLÊNCIA E DISCRIMINAÇÃO NO AMBIENTE UNIVERSITÁRIO

QUESTÃO	CATEGORIAS DE RESPOSTAS	CÓDIGOS	PONTOS	OBSERVAÇÃO
17. Nesta posição que agora ocupa, com qual frequência você foi informado (a) sobre alguma situação de violência que ocorrem sua unidade?	Frequentemente<br>Às vezes<br>Raramente<br>Nunca	1<br>2<br>3<br>4	4<br>3<br>2<br>1	
18. Esta violência ocorreu entre: (leia as alternativas)<br>Entre professores e estudantes<br>Entre estudantes e estudantes<br>Entre funcionários e funcionários<br>Entre professores e professores<br>Entre funcionários e estudantes<br>Entre funcionários e professores	Mesmas opções para todas as manifestações<br>Sim<br>Não	 1<br>0	 1<br>0	Cada manifestação afirmativa recebe 1 ponto. A somatória dos pontos das categorias define um escore de 0 a 6 pontos
19. Qual o tipo de violência você foi informado? (leia as alternativas)<br>Física<br>Psicológica<br>Sexual<br>Assédio moral<br>Assédio sexual<br>Outro tipo de violência<br>Qual?	Mesmas opções para todas as manifestações<br>Sim<br>Não<br>Campo texto	 1<br>0	 1<br>0	Cada manifestação afirmativa recebe 1 ponto. A somatória dos pontos das categorias define um escore de 0 a 6 pontos
20. Nesta posição que agora ocupa, você foi informado sobre alguma situação de discriminação que ocorre em sua unidade por algum desses motivos? (leia as alternativas)<br>Sexo<br>Idade<br>Orientação sexual<br>Raça/cor<br>Origem social<br>Outro motivo<br>Qual outro motivo?	Mesmas opções para todas as manifestações<br>Sim<br>Não<br>Campo texto	 1<br>0	 1<br>0	Cada manifestação afirmativa recebe 1 ponto. A somatória dos pontos das categorias define um escore de 0 a 6 pontos
21. Com que frequência você consulta o Regimento Geral da USP?	Frequentemente<br>Às vezes<br>Raramente<br>Nunca	1<br>2<br>3<br>4	1<br>2<br>3<br>3	
22. Antes de ocupar esta sua posição atual você	Mesmas opções para todas as manifestações			Cada manifestação

presenciou pelo menos uma situação que considerou violenta na universidade? A violência ocorreu entre: (leia as alternativas)<br>Entre professores e estudantes<br>Entre estudantes e estudantes<br>Entre funcionários e funcionários<br>Entre professores e professores<br>Entre funcionários e estudantes<br>Entre funcionários e professores	Sim<br>Não	1<br>0	1<br>0	afirmativa recebe 1 ponto. A somatória dos pontos das categorias define um escore de 0 a 6 pontos
23. Antes de ocupar esta sua posição atual, qual (is) foi (foram) o (os) tipo (s) de violência presenciado (s) por você? (leia as alternativas)<br>Física<br>Psicológica<br>Sexual<br>Assédio moral<br>Assédio sexual<br>Outro tipo de violência<br>Qual?	Mesmas opções para todas as manifestações<br>Sim<br>Não<br>Campo texto	1<br>0	1<br>0	Cada manifestação afirmativa recebe 1 ponto. A somatória dos pontos das categorias define um escore de 0 a 6 pontos
23.a. Quando isto ocorreu você era:	Estudante<br>Docente<br>Funcionário<br>Docente e estudante	1<br>2<br>3<br>4		Não pontua, porque é vinculada à resposta da questão 23 e é somente descritiva
23.b. Você pode dizer se esta ocorrência era: (leia as alternativas)	Frequentemente<br>Às vezes ocorria<br>Rara	1<br>2<br>3		Não pontua, porque é vinculada à resposta da questão 23 e é somente descritiva

24. Você já foi discriminado na sua profissão por alguma desses motivos? (leia as alternativas)<br>Sexo<br>Idade<br>Orientação sexual<br>Raça/cor<br>Origem social<br>Outro motivo<br>Origem acadêmica<br>Qual outro motivo?	Mesmas opções para todas as manifestações<br>Sim<br>Não<br>Campo texto	1<br>0	1<br>0	Cada manifestação afirmativa recebe 1 ponto. A somatória dos pontos das categorias define um escore de 0 a 7 pontos
25. Antes de ocupar esta sua posição atual, você já	Mesmas opções para todas as manifestações			Cada manifestação

presenciou pelo menos uma situação que considerou discriminatória na universidade? A discriminação ocorreu entre: (leia as alternativas)<br>Entre professores e estudantes<br>Entre estudantes e estudantes<br>Entre funcionários e funcionários<br>Entre professores e professores<br>Entre funcionários e estudantes<br>Entre funcionários e professores	Sim<br>Não	1<br>0	1<br>0	afirmativa recebe 1 ponto. A somatória dos pontos das categorias define um escore de 0 a 6 pontos
26. Antes de ocupar esta sua posição atual, qual (is) foi (foram) o (os) tipo (s) de discriminação presenciado (s) por você? (leia as alternativas)<br>Sexo<br>Idade<br>Orientação sexual<br>Raça/cor<br>Origem social<br>Outro motivo<br>Minoria<br>Qual outro motivo?	Mesmas opções para todas as manifestações<br>Sim<br>Não<br>Campo texto	1<br>0	1<br>0	Cada manifestação afirmativa recebe 1 ponto. A somatória dos pontos das categorias define um escore de 0 a 7 pontos
26.a. Quando isto ocorreu você era: (leia as alternativas)	Estudante<br>Docente<br>Funcionário<br>Docente e estudante	1<br>2<br>3<br>4		Não pontua, porque é vinculada à resposta da questão 26 e é somente descritiva
26.b.Você pode dizer se esta ocorrência era: (leia as alternativas)	Frequentemente<br>Às vezes ocorria<br>Rara	1<br>2<br>3		Não pontua, porque é vinculada à resposta da questão 26 e é somente descritiva
27. Quando você era estudante você participava do trote?	Não<br>Sim	0<br>1		Não pontua, porque é somente descritiva
27.a. Na época em que você era estudante, durante o ingresso na universidade existiam as seguintes situações?<br>Apelidos<br>Xingamentos<br>Humilhações<br>Coerção para beber<br>Agressões físicas	Mesmas opções para todas as manifestações<br>Sim<br>Não	1<br>0	1<br>0	Cada manifestação afirmativa recebe 1 ponto. A somatória dos pontos das categorias define um escore de 0 a 5 pontos

Parte 5. SEÇÃO CONHECIMENTO SOBRE PROCEDIMENTOS RELATIVOS À VIOLÊNCIA DE GÊNERO NO AMBIENTE UNIVERSITÁRIO

QUESTÃO	CATEGORIAS DE RESPOSTAS	CÓDIGOS	PONTOS	OBSERVAÇÃO
28. Você considera que a violência em função de gênero se refere exclusivamente à mulher?	Sim	1	0	Categoria 2 (não) é a resposta correta
	Não	2	1	
	Não sabe	3	0	
28.a.Na sua formação, você teve algum contato com conteúdos relativos à violência de gênero?	Não	0		Não pontua. É variável de contexto
	Sim	1		
29. Você acha que a porcentagem de mulheres no Brasil que sofreram algum tipo de violência nos últimos 12 meses é cerca de:	Entre 10 e 15%	1	0	Categoria 2 (Entre 25 e 30 %) é a resposta correta. 30% a 40% foi retirado propositalmente
	Entre 25 e 30 %	2	1	
	Entre 50 e 60%	3	0	
	Entre 75 e 80%	4	0	
	Não sabe	5	0	
30. O que acontece no cotidiano envolvendo a violação de direitos humanos nas interações na comunidade acadêmica não é responsabilidade da universidade e seus gestores.	Verdadeiro	1	0	Categoria 2 (falso) é a resposta correta
	Falso	2	1	
	Não sabe	3	0	
31. Mecanismos administrativos previstos no Regimento Geralda Universidade não garantem a proteção e atendimento integral às vítimas de violência de gênero.	Verdadeiro	1	0	Categoria 2 (falso) é a resposta correta
	Falso	2	1	
	Não sabe	3	0	
32. Se a violência contra a mulher acontecer em uma festa de república, por exemplo, a universidade e seus gestores nada podem fazer, pois escapa ao ambiente universitário	Verdadeiro	1	0	Categoria 2 (falso) é a resposta correta
	Falso	2	1	
	Não sabe	3	0	
33. Em caso de queixa de violência de gênero em uma unidade USP, o poder disciplinar em uma unidade da USP deve ser exercido por quem?<br>Reitor<br>Diretor ou Chefe de Departamento<br>Prefeito do Campus<br>Congregação	Mesmas opções para todas as manifestações			Categoria 1 (sim) é a resposta esperada para cada manifestação. A somatória dos pontos das categorias define um escore de 0 a 6 pontos
	Sim	1	1	
	Não	2	0	
	Não sabe	3	0	

Outros<br>Comissões especiais<br>Outros quem?	Campo texto			
34. Na sua opinião, ao tomar conhecimento de algum caso de violência de gênero qual seria a conduta ou as condutas mais correta(s) para o gestor universitário tomar? (Não ler as alternativas)<br>a) Instaurar ou pedir para que a autoridade competente instaure uma sindicância para apurar a situação.<br>b) Garantir que as partes sejam ouvidas na comissão sindicante.<br>c) Orientar a vítima a registrar a ocorrência na Delegacia de Defesa da Mulher (DDM).<br>d) Acolher a vítima<br>e) Providenciar para que a vítima seja acolhida por meio de escuta qualificada<br>f) Orientar a vítima quanto aos seus direitos e os procedimentos a serem tomados seguirão a escolha compartilhada.<br>g) Encaminhar a situação à ouvidoria da universidade para providências cabíveis.<br>h) Não sabe	Opções para as alternativas "a" até "f"<br>Não<br>Sim	 0<br>1	 0<br>1	Categoria 1 (sim) é a resposta correta
	Opções para as alternativas "g" e "h"<br>Não<br>Sim	 1<br>0	 1<br>0	Categoria 1 (não) é a resposta correta<br><br>A somatória dos pontos das categorias define um escore de 0 a 8 pontos
Outra. Qual?	Campo texto			
35. A composição de uma comissão sindicante para apurar uma situação de violência contra mulher deve manter uma perspectiva de gênero, e, nesse sentido, evitar qualquer tipo de estereótipo, preconceito ou discriminação de gênero	Verdadeiro<br>Falso<br>Não sabe	1<br>2<br>3	1<br>0<br>0	Categoria 1 (verdadeiro) é a resposta correta
36. É responsabilidade institucional da universidade enfrentar as situações de violência de gênero, de acordo com o que está previsto na legislação atual, como a Lei Maria da Penha.	Verdadeiro<br>Falso<br>Não sabe	1<br>2<br>3	1<br>0<br>0	Categoria 1 (verdadeiro) é a resposta correta
37. Aos membros de uma comissão sindicante, cabe	Verdadeiro<br>Falso	1<br>2	1<br>0	

ouvir as partes em separado, não emitir qualquer juízo de valor e nunca questionar as condutas notificadas	Não sabe	3	0	Categoria 1 (verdadeiro) é a resposta correta
38. Mulheres em situação de violência têm direito a um atendimento integral e é dever da Instituição de Ensino Superior.	Verdadeiro<br>Falso<br>Não sabe	1<br>2<br>3	1<br>0<br>0	Categoria 1 (verdadeiro) é a resposta correta

Parte 6. INFORMAÇÕES SOCIODEMOGRÁFICAS DOS PARTICIPANTES

QUESTÃO	CATEGORIAS DE RESPOSTAS	CÓDIGOS	PONTOS	OBSERVAÇÃO
A. Data de nascimento	formato dd mm aaaa	--	--	Não pontua no escore
B. Idade em anos completos	Quantitativa discreta	--	--	Não pontua no escore
C. Sexo	Feminino<br>Masculino	--<br>--	--<br>--	Não pontua no escore
D. Raça/cor, por autoidentificação, de acordo com a classificação do IBGE	Preta<br>Parda<br>Indígena<br>Branca<br>Amarela	--<br>--<br>--<br>--<br>--	--<br>--<br>--<br>--<br>--	Não pontua no escore
E. Religião, de acordo com a classificação do IBGE	Católica (apostólica romana; romana; apostólica brasileira e ortodoxa)<br>Evangélica pentecostal/neopentecostal (ass. de Deus; Congregação Cristã; Brasil para Cristo; evangelho quadrangular; Universal; Casa da Benção; Deus é Amor; Maranata; Nova Vida; Renovada; Comunidade Evangélica; etc.)<br>Evangélica de missão ou outra denominação (Adventista; Adventista do Sétimo Dia; Batista; Congregacional; Luterana; de Missão Luterana; Presbiteriana; Metodista, etc.)<br>Espírita<br>Umbandista ou candomblecista<br>Sem religião (se a pessoa responder "não tenho")<br>Outra<br>Qual outra (campo texto)	--<br>--<br>--<br>--<br>--<br>--<br>--<br>--	--<br>--<br>--<br>--<br>--<br>--<br>--<br>--	Não pontua no escore
F. Prática semanal da religião	Sim<br>Não	--<br>--	--<br>--	Não pontua no escore
G. Possui filhos	Sim<br>Não	--<br>--	--<br>--	Não pontua no escore
H. Número de filhos	Quantitativa discreta	--	--	Não pontua no escore
I. Sexo dos filhos	Feminino<br>Masculino<br>Ambos	--<br>--<br>--	--<br>--<br>--	Não pontua no escore

J. Orientação sexual	Oposto<br>O mesmo que eu<br>Ambos<br>Prefere não falar	--<br>--<br>--<br>--	--<br>--<br>--<br>--	Não pontua no escore
K. Quer falar mais algo em relação ao tema da pesquisa	Campo texto	--	--	Não pontua no escore
L. O que a pessoa pensa que poderia ser feito na universidade para melhorar o relacionamento entre as pessoas	Campo texto	--	--	Não pontua no escore
M. Número de telefone da pessoa, para verificação do questionário	Campo texto	--	--	Não pontua no escore
