# Supplementary material for: Psychometric Properties of an Instrument for Assessing University Administrators’ Knowledge on Gender-Based Violence
Source: Rev Bras Enferm. 2023 Dec 4;76(6):e20220770. doi: 10.1590/0034-7167-2022-0770 (PMC10695057; doi:10.1590/0034-7167-2022-0770)
Supplement: 0034-7167-reben-76-06-e20220770-suppl02 [file 0034-7167-reben-76-06-e20220770-suppl02.pdf]

Tabela 1 – Análise descritiva das características pessoais e ocupacionais dos gestores universitários, Universidade de São Paulo campus Ribeirão Preto, setembro a novembro de 2020, Ribeirão Preto.

Variável	Nº	%
**Sexo**		
Feminino	51	50,5
Masculino	50	49,5
**Faixa etária**		
33 a 49 anos	37	36,6
50 a 59 anos	46	45,5
60 a 74 anos	18	17,8
**Raça/cor**		
Preta ou parda	16	15,8
Branca	82	81,2
Amarela	3	3,0
**Religião**		
Católica	50	49,5
Evangélica pentecostal	3	3,0
Espírita	9	8,9
Sem religião	32	31,7
Outra religião	7	6,9
**Estado Civil**		
Casado (a)	72	71,3
Em união estável	12	11,9
Solteiro (a) ou divorciado (a)	17	16,8
**Tem filhos**		
Sim	78	77,2
Não	23	22,8
**Orientação sexual**		
Heterossexual	96	95,0
Homossexual	3	3,0
Não quis falar	2	2,0
**Tempo de trabalho na universidade**		
Até 18 anos	51	50,5
Mais de 18 anos	50	49,5
**Cargo atualmente ocupado**		
Diretores, vice-diretores e presidentes de comissões estatutárias	46	45,5
Presidentes de CoCs, chefes e vice-chefes de departamento	39	38,6
Assistência técnica ou representante de funcionários na congregação/CTA	16	15,8
**Tempo de gestão no cargo**		
Abaixo de um ano	33	32,7
De um ano até dois anos	30	29,7
Acima de dois anos	38	37,6
**Teve cargo de gestão anterior**		
Sim	87	86,1

Não	14	13,9
**Titularidade ou posição na carreira**		
Professor Doutor 1 ou 2	16	15,8
Professor Associado 1, 2 ou 3	42	41,6
Professor Titular	27	26,7
Servidor de qualquer nível/posição	16	15,8
**Total**	**101**	**100,0**
