# Supplementary material for: Psychometric Properties of an Instrument for Assessing University Administrators’ Knowledge on Gender-Based Violence
Source: Rev Bras Enferm. 2023 Dec 4;76(6):e20220770. doi: 10.1590/0034-7167-2022-0770 (PMC10695057; doi:10.1590/0034-7167-2022-0770)
Supplement: 0034-7167-reben-76-06-e20220770-suppl03 [file 0034-7167-reben-76-06-e20220770-suppl03.pdf]

MATERIAL SUPLEMENTAR 2 – QUESTIONÁRIO FINAL APÓS ANÁLISE FATORIAL

Questionário para Avaliação do Conhecimento dos Gestores Universitários sobre Violência na Universidade"

No. Original	Questão Final	Categorias De Respostas	Código	Ponto	Obs	Fator 1*	Fator 2*
4	1. Qual é a frequência da violência no ambiente universitário?	Frequentemente<br>Às vezes<br>Raramente<br>Nunca<br>Não sabe	1<br>2<br>3<br>4<br>5	0<br>1<br>0<br>0<br>0	Categoria 2 (às vezes) é a resposta correta	**X**	
5	2. Qual é a frequência do machismo e da discriminação sexual no ambiente universitário?	Frequentemente<br>Às vezes<br>Raramente<br>Nunca<br>Não sabe	1<br>2<br>3<br>4<br>5	0<br>1<br>0<br>0<br>0	Categoria 2 (às vezes) é a resposta correta	**X**	
	**Nas próximas seis perguntas agora eu vou ler algumas afirmações e você vai me dizer se você concorda plenamente, concorda, não concorda nem discorda, discorda e discorda plenamente. Vou mostrar as opções para você na tela**						
11	3. Casos de violência que possam acontecer em festas, repúblicas e torneios fora do campus, entre universitários, não devem ser considerados no âmbito da violência universitária.	Concordo plenamente<br>Concordo<br>Não concordo e nem discordo<br>Discordo<br>Discordo totalmente	1<br>2<br>3<br>4<br>5	1<br>2<br>0<br>3<br>4	Quanto menor a pontuação, maior a tolerância à violência	**X**	
12	4. Pintar o corpo, fazer pedágio, cortar o cabelo e usar adereços não deve ser considerado trote pois não envolve violência entre os estudantes.	Concordo plenamente<br>Concordo<br>Não concordo e nem discordo<br>Discordo<br>Discordo totalmente	1<br>2<br>3<br>4<br>5	1<br>2<br>0<br>3<br>4	Quanto menor a pontuação, maior a tolerância à violência		**X**
14	5. Brincadeiras entre veteranos e calouros, que incluem apelidos engraçados, pagar micos,	Concordo plenamente<br>Concordo<br>Não concordo e nem discordo<br>Discordo	1<br>2<br>3<br>4	1<br>2<br>0<br>3	Quanto menor a pontuação, maior a tolerância à violência		**X**

	fazer pedágios, etc., fazem parte da cultura universitária e não devem ser consideradas trotes.	Discordo totalmente	5	4			
15	6. Homens e mulheres são tratados igualmente por todos no ambiente universitário.	Concordo plenamente<br>Concordo<br>Não concordo e nem discordo<br>Discordo<br>Discordo totalmente	1<br>2<br>3<br>4<br>5	1<br>2<br>0<br>3<br>4	Quanto menor a pontuação, maior a tolerância à violência	X	
16	7. A universidade está preparada para enfrentar a discriminação de classe, sexo, gênero e raça entre seus membros.	Concordo plenamente<br>Concordo<br>Não concordo e nem discordo<br>Discordo<br>Discordo totalmente	1<br>2<br>3<br>4<br>5	1<br>2<br>0<br>3<br>4	Quanto menor a pontuação, maior a tolerância à violência	X	
17	8. Nesta posição que agora ocupa, com qual frequência você foi informado (a) sobre alguma situação de violência que ocorre em sua unidade?	Frequentemente<br>Às vezes<br>Raramente<br>Nunca	1<br>2<br>3<br>4	4<br>3<br>2<br>1			X
18	9. Esta violência ocorreu entre: (leia as alternativas)<br>Entre professores e estudantes<br>Entre estudantes e estudantes<br>Entre funcionários e funcionários<br>Entre professores e professores<br>Entre funcionários e estudantes	Mesmas opções para todas as manifestações<br>Sim<br>Não	 1<br>0	 1<br>0	Cada manifestação afirmativa recebe 1 ponto. A somatória dos pontos das categorias define um escore de 0 a 6 pontos		X

	Entre funcionários e professores						
19	10. Qual o tipo de violência você foi informado? (leia as alternativas)<br>Física<br>Psicológica<br>Sexual<br>Assédio moral<br>Assédio sexual<br>Outro tipo de violência<br>Qual?	Mesmas opções para todas as manifestações<br>Sim<br>Não<br>Campo texto	 1<br>0	 1<br>0	Cada manifestação afirmativa recebe 1 ponto. A somatória dos pontos das categorias define um escore de 0 a 6 pontos		X
20	11. Nesta posição que agora ocupa, você foi informado sobre alguma situação de discriminação que ocorre em sua unidade por algum desses motivos? (leia as alternativas)<br>Sexo<br>Idade<br>Orientação sexual<br>Raça/cor<br>Origem social<br>Outro motivo<br>Qual outro motivo?	Mesmas opções para todas as manifestações<br>Sim<br>Não<br>Campo texto	 1<br>0	 1<br>0	Cada manifestação afirmativa recebe 1 ponto. A somatória dos pontos das categorias define um escore de 0 a 6 pontos		X
22	12. Antes de ocupar esta sua posição atual você presenciou pelo menos uma situação que considerou violenta na universidade? A violência ocorreu entre: (leia as alternativas)<br>Entre professores e estudantes<br>Entre estudantes e estudantes	Mesmas opções para todas as manifestações<br>Sim<br>Não	 1<br>0	 1<br>0	Cada manifestação afirmativa recebe 1 ponto. A somatória dos pontos das categorias define um escore de 0 a 6 pontos	X	

	Entre funcionários e funcionários<br>Entre professores e professores<br>Entre funcionários e estudantes<br>Entre funcionários e professores					
23	13. Antes de ocupar esta sua posição atual, qual (is) foi (foram) o (os) tipo (s) de violência presenciado (s) por você? (leia as alternativas)<br>Física<br>Psicológica<br>Sexual<br>Assédio moral<br>Assédio sexual<br>Outro tipo de violência<br>Qual?	Mesmas opções para todas as manifestações<br>Sim<br>Não<br>Campo texto	1<br>0	1<br>0	Cada manifestação afirmativa recebe 1 ponto. A somatória dos pontos das categorias define um escore de 0 a 6 pontos	X
24	14. Você já foi discriminado na sua profissão por alguma desses motivos? (leia as alternativas)<br>Sexo<br>Idade<br>Orientação sexual<br>Raça/cor<br>Origem social<br>Outro motivo<br>Origem acadêmica<br>Qual outro motivo?	Mesmas opções para todas as manifestações<br>Sim<br>Não<br>Campo texto	1<br>0	1<br>0	Cada manifestação afirmativa recebe 1 ponto. A somatória dos pontos das categorias define um escore de 0 a 7 pontos	X
25	15. Antes de ocupar esta sua posição atual, você já presenciou pelo menos uma situação que considerou discriminatória na universidade?	Mesmas opções para todas as manifestações<br>Sim<br>Não	1<br>0	1<br>0	Cada manifestação afirmativa recebe 1 ponto. A somatória dos pontos das categorias define um	X

	A discriminação ocorreu entre: (leia as alternativas)<br>Entre professores e estudantes<br>Entre estudantes e estudantes<br>Entre funcionários e funcionários<br>Entre professores e professores<br>Entre funcionários e estudantes<br>Entre funcionários e professores				escore de 0 a 6 pontos		
26	16. Antes de ocupar esta sua posição atual, qual (is) foi (foram) o (os) tipo (s) de discriminação presenciado (s) por você? (leia as alternativas)<br>Sexo<br>Idade<br>Orientação sexual<br>Raça/cor<br>Origem social<br>Outro motivo<br>Minoria<br>Qual outro motivo?	Mesmas opções para todas as manifestações<br>Sim<br>Não<br>Campo texto	1<br>0	1<br>0	Cada manifestação afirmativa recebe 1 ponto. A somatória dos pontos das categorias define um escore de 0 a 7 pontos	**X**	**X**
34	17. Na sua opinião, ao tomar conhecimento de algum caso de violência de gênero qual seria a conduta ou as condutas mais correta(s) para o gestor universitário tomar? (Não ler as alternativas)	Opções para as alternativas "a" até "f"<br>Não<br>Sim	0<br>1	0<br>1	Categoria 1 (sim) é a resposta correta		**X**

	a) Instaurar ou pedir para que a autoridade competente instaure uma sindicância para apurar a situação.<br>b) Garantir que as partes sejam ouvidas na comissão sindicante.<br>c) Orientar a vítima a registrar a ocorrência na Delegacia de Defesa da Mulher (DDM).<br>d) Acolher a vítima<br>e) Providenciar para que a vítima seja acolhida por meio de escuta qualificada<br>f) Orientar a vítima quanto aos seus direitos e os procedimentos a serem tomados seguirão a escolha compartilhada.<br>g) Encaminhar a situação à ouvidoria da universidade para providências cabíveis.<br>h) Não sabe	Opções para as alternativas "g" e "h"<br>Não<br>Sim	 1<br>0	 1<br>0	Categoria 1 (não) é a resposta correta<br>A somatória dos pontos das categorias define um escore de 0 a 8 pontos	
	Outra. Qual?	Campo texto				
37	18. Aos membros de uma comissão sindicante, cabe ouvir as partes em separado, não emitir qualquer juízo de valor e nunca questionar as	Verdadeiro<br>Falso<br>Não sabe	1<br>2<br>3	1<br>0<br>0	Categoria 1 (verdadeiro) é a resposta correta	X

	condutas notificadas						
38	19. Mulheres em situação de violência têm direito a um atendimento integral e é dever da Instituição de Ensino Superior.	Verdadeiro<br>Falso<br>Não sabe	1<br>2<br>3	1<br>0<br>0	Categoria 1 (verdadeiro) é a resposta correta		X

* Fator 1 = Conhecimento/experiência atual
Fator 2 = Conhecimento/experiência pregresso
